# Commissions

**Published:** 1898-12-24

**Authors:** 


### COMMISSIONS.

Christmas being the great season for tips and commissions, things which, even under the guise of Christmas boxes, are full of offence to persons whose ethics are of proper mould, we may refer once more to the extremely derogatory form that the "commission system," in regard to professional affairs, has taken on at the other side of the Atlantic. We do not ourselves believe that the canker has spread very far, for among our brethren in America there are men as honourable as are anywhere to be found. Still, unless Americans are greatly belied by their own journals, we can hardly doubt that it exists, and that it has taken a hold in some professional quarters, which is most lamentable. The position seems to be that when a practitioner meets with a case which requires consultation or operation, instead of seeking the specialist who will best do what is wanted, he hunts around to find the one who will give him the largest commission. The New York *Medical Record,* writing about this abominable custom, says: "What has been called 'bargain counter' surgery, especially in the realms of abdominal and pelvic pathology, must have some existence in fact, or one would not hear of it from so many different sources," and enforces its point by the following interesting little story—"It was some years ago that many reputable surgeons in this city were shocked in their finer susceptibilities by a down East doctor who landed in town with six laparotomy cases, which he proceeded to farm out on terms most advantageous to himself. Many of the surgeons approached had not before heard of such shrewd, businesslike methods being applied to human existence, and very properly showed the visitor the door. Undoubtedly these surgeons would pursue the same course to-day. It is, at least, to be hoped they would. Whether or not this hawkster succeeded in finding an operator sufficiently devoid of ethical honesty to offer the proper 'divy' is not divulged." The law as to commissions has lately been pretty clearly laid down in this country, and we hope that, if any of these little tricks are discovered, the perpetrators will be given short shrift, not only by way of professional ostracism, but by those less gentle processes by which the law courts enforce their judgments.

